# Governing Globalisation through Law

**DOI:** 10.1017/err.2020.31

**Published:** 2020-04-30

**Authors:** Mireille DELMAS-MARTY

**Affiliations:** * Member of Institut de France and Emeritus Professor at Collège de France; email: hugo.pascal@sciencespo.fr.

## I. Introduction: building the rule of law without a global state

Governing globalisation through law implies building the rule of law without a world State, and therefore rethinking the tool that law, traditionally identified with the State, represents in the face of the interdependencies born of globalisation and the challenges they generate. Economic and financial crises, social crises, global terrorism; the humanitarian disaster of migrations, the climate crisis and, to top it all off, the coronavirus health crisis: it is time to take them seriously, as the cacophony of this poly-crisis amplifies. As if citizen indignation at security abuses, the anger of the yellow vests at social inequalities, the revolt of the younger generations and the calls of scientists regarding climate change had not been enough, it took a simple virus, smaller than a butterfly’s wing, to shake the world, to the point of finally shaking the certainties of our leaders.

The great powers, or those merely thinking of themselves as such, proud of their new technologies and convinced of their political and/or economic power, are proving incapable of coordinating on a global scale. It is as if this tiny living being had come as a messenger to challenge our globalised humanity and reveal its fragility, offering it one last chance to realise its common destiny. In sum, a human commitment to better govern the galloping and unpredictable globalisation is needed.

## II. Between sovereignism and universalism

Our concept of sovereignty needs to be renewed. In order to create the rule of law without a true world State, universalism is too ambitious, and sovereignism, by retreating into national communities, is too weak. Reconciling sovereignism and universalism requires thinking about them interactively. It is not a matter of choosing between the two, but of combining them in order to reconcile them. This is why we still need national communities to hold accountable the main actors of globalisation (States and transnational corporations (TNCs)), but only the global community will be able to define common objectives and the resulting responsibilities. And only their interweaving will prevent the two dynamics from opposing and neutralising each other, leading to a society of “unlimited irresponsibility”.

It is therefore necessary for sovereignism to be “internationalised”, as national legislations incorporate international agreements. For example, in environmental matters, national legislation is interpreted in light of national states’ international commitments or, more broadly, in light of the international conventions put in place. At the same time, this process of internationalisation is leading national judges, in this case National judges, to become European or even global judges when they directly apply European or international standards.

The opposite phenomenon is also necessary because universalism, in order to be applicable in the real world, needs to be “contextualised”. The legal technique of harmonisation makes it possible, without going as far as unification, to give a concrete form to the idea of “ordering pluralism”.^1^ In order to achieve this, the European Court of Human Rights (ECHR) allows a “national margin of appreciation” on sensitive issues, such as the ban on wearing the full veil in places open to the public. To uphold respect for privacy, freedom of religion and freedom of expression, the “restrictions necessary in a democratic society” clause allows the Court to grant national judges a “national margin” of appreciation, but only a margin that does not allow a complete re-nationalisation. If a national decision let a State exceed the limits of compatibility, it will be overturned.

In economic and financial matters, we find the same idea of a “contextualised” universalism with the formula of “common and differentiated responsibilities”, such as in the past decisions of the Dispute Settlement Body (DSB), the World Trade Organization’s (WTO) appellate body or the Kyoto Protocol or the Paris Climate Agreement.

What seems to emerge from these international agreements is the idea of a multiple commonality, at the crossroads between uniformity and plurality, found in the notion of “functional equivalence”.^2^ This notion also takes into account the empirical reality that each legal system has its own logic and context. Beyond normative and institutional comparisons, it makes it possible to assess whether the effects produced by a national legal system meet the requirements set by an international agreement. Especially suited to the procedure of peer evaluation, this notion is used in particular in the fight against international corruption.^3^

The result is an imperfect harmonisation between sovereignism and universalism, a convergence through a kind of “legal tinkering”. There is no genuine law of globalisation that would be perfectly coherent and neither national nor international. In practice, to paraphrase the biologist François Jacob’s formula describing the evolution of life, jurists make “new with old”.^4^ In other words, they make “new” by reinterpreting old law, national or international. By crossing these ancient forms, they replace binary logic with more complex forms such as “internationalised” sovereignty, or “contextualised” universalism. This is why lawyers are led to think through complexity.

## III. Thinking through complexity

Of course, it would be possible to govern globalisation through law in a simple way. It would suffice to set up a hegemonic system by extending the legislation of the most powerful country to the rest of the world. There has been the American attempt regarding financial crimes, and one can glimpse the Chinese dream over the horizon of the “New Silk Roads”.^5^ Yet so far no empire has functioned on a planetary scale.

The traditional image to which legal thought refers is that of the pyramid of norms, built by each State in a linear, hierarchical and static way. However, in a globalised world, the legal system is plural, interactive, combinatorial and evolutionary, because it is built from interactions that are based on alternative logics, such as fuzzy logic. This logic, which handles the concept of partial truth, consists in assessing the degree of proximity of a practice to the reference norm. A careful study of the judgements of the ECHR shows that fuzziness is not always synonymous with arbitrariness and inconsistency: it is possible to construct rational and predictable reasoning with fuzzy concepts, provided that the judge makes an effort to be transparent, in clarifying their criteria, and rigorous, in applying the same criteria with the same weight from one case to another. Thus formalised, fuzzy logic makes it possible to adapt legal reasoning to situations too imprecise to be conceived in binary logic.

The autonomous notion of “criminal matter” is a striking example. Whereas “criminal” is traditionally what the legislature has so defined, the ECHR has developed the idea that the guarantees of the legal regime specific to criminal law can be extended to areas related to “criminal matters”. However, the judge still has to explain the criteria justifying the application of this concept (transparency). What makes a norm or sanction sufficiently close to criminal law to require compliance with more demanding rules (legality, non-retroactivity, etc.)? The Court has laid down several criteria, such as the severity of the sanction or the generality of the offence. Fuzzy logic becomes necessary in a legal universe that is all the more imprecise as it becomes more global. But it implies a transfer of power to the interpreter (the judge or a similar body) and will only be predictable if the motivation is transparent and the reasoning rigorous.

It should be added that fuzziness is sometimes accompanied by a non-binding and non-sanctioned *soft law*. In contrast, *hard law* is precise, binding and sanctioned. Less constraining at first sight, *soft law* is sometimes more effective, and ultimately more repressive, than *hard law*.^6^

Compliance provides a good example of this, because the settlement mechanism (*plea deal*)^7^ at the origin of compliance constitutes a relaxation of American criminal law. It offers the possibility, instead of initiating criminal proceedings, of proceeding to a negotiation between prosecution and defence in order to dismiss criminal charges. The prosecutor saves years of searching for evidence, while the accused (often top executives) avoids a long trial that can result in very heavy prison sentences and permanent damage to the company’s image. This is in everyone’s interest, including the US Treasury, which collects fines amounting to billions of dollars.

Corporate social responsibility provides another example of the interaction between *soft law* and *hard law*. Initially, responsibility meant participation in company decisions, but without the duty to account for them. However, this *soft law* has become harder over time because of the consequences attached to corporate commitments. Commitments, even spontaneous and voluntary, became actionable against companies in order to hold them liable, this time legally. Several offences, such as misleading advertising or some labour code offences, which trigger civil liability proceedings, allow for transformative lawsuits.

The same is true of the new “climate lawsuits”, filed not only against States, but also against companies. The French statute on the duty of vigilance,^8^ adopted in 2017, following the collapse of the Rana Plaza textile workshop in Bangladesh, contributes to this tightened accountability. As a way of toughening *soft law*, this statute imposes on companies the duty to check with subsidiaries and subcontractors all along the value chain.

Finally, even if one does not yet measure the disruption that could result from the new PACTE statute of 2019^9^ on the *raison d’être* of companies and the expanded corporate interest, it can be estimated that the hardening of these notions could become one of the processes contributing to the establishment of an economy of public goods,^10^ and a new anthropology is emerging between humanity and nature.

## IV. A global governance aggregating Knowledge, Will and Power

In the conclusion of the book *Aux quatre vents du monde*,^11^ I transposed a poem by Edouard Glissant entitled “Au congrès des vents”, imagining a kind of congress bringing together all of the major actors of globalisation, each one envisioning himself as “master of the winds”. Then came a “little unnamed wind from the countryside”. As a citizen of the world, whose vital impetus represents the new generations, it asserts “we don’t need a master of the winds”, because it will either be powerless or turn into a tyrant. It is better that everyone takes responsibility for a share of the common goods. In order to preserve these goods, we therefore need a constellation of actors, both public and private.

States cannot be the only public actors. Alongside them, local and regional authorities, already organised in networks, contribute to structuring globalisation horizontally. Judges and public prosecutors (national and international) will undoubtedly play the role of third parties guaranteeing impartiality, an essential condition of the rule of law that lawyers will help to invigorate. In some domains, such as economic law or digital law, the Court of Justice of the European Union (CJEU) has even become a hub of global regulation and has already made a major contribution to the conceptualisation of a common law. However, it cannot be the only institution in charge of democratic control. It is important in Europe to have two supreme courts: a human rights court and a court destined originally to the “common market”. Although it now has incorporated the fundamental rights enshrined in the European Charter of 2000, the CJEU does not replace the ECHR in human rights matters. Maintaining this bipolarity (market/human rights) with two courts (CJEU and ECHR) is perhaps one of the keys to a dynamic balance.

At the global level, one can imagine a similar balance between the WTO’s appellate body, the International Labour Organization’s (ILO) expert panel, provided its control over social rights is strengthened, as well as the UN Human Rights Committee. One could even imagine a Common Goods Court responsible for ensuring coherence of the whole, unless the International Court of Justice becomes sufficiently autonomous to play this role. In any case, excessive sectorisation of international law must be avoided.

As for implementation, the role of national prosecutors should not be neglected, as they might be tempted to take their cue from the American prosecutors’ practices for financial offences falling within the extraterritorial application of US law. At the international level, mention should be made of the International Criminal Court (ICC) prosecutor for the “most serious” international crimes. The newly created European public prosecutor could be part of this constellation, provided that they are powerful enough to be effective, like the American prosecutor, who remains a reference. Yet, this European public prosecutor, as the Member States wanted it, lost the autonomy contemplated by the expert group I chaired (*Corpus Juris*, 1998^12^), which desired to endow the office with a certain autonomy vis-à-vis the Member States. The European public prosecutor’s office, which finally came into operation in 2020, is much less autonomous. Only the public prosecutor and their deputy have European status. As a result, the European prosecutor’s office remains dependent on national legislations and statuses specific to each European country. Although it risks being weakened, the very existence of a European prosecutor is a step forward, and its future will also depend on how the role is played in the first few months of operation: a high level of competence and a strong charismatic authority could make up for this legal weakness.

Finally, an important role in the regulation of globalisation is played by civic actors (ie citizens, non-governmental organisations, associations and trade unions). Civil society is even broader, since it includes private economic actors (TNCs have become real powers competing with States) and scientific actors, whose knowledge is sometimes decisive, especially in a field such as climate change. The alliance between (scientific) knowledge and (civic) will should make it possible to oversee (political and economic) powers.

At the European level, and *a fortiori* at the global level, the classical theory of the separation of powers cannot be directly transposed, if only because there is no global executive power, nor a global legislator. On the other hand, jurisdictions are involved in global governance, even when their status remains linked to the national framework. Montesquieu’s theory is therefore not transposable, as it would presuppose a global State, which is neither feasible nor desirable. Thus, we must seek to transpose the democratic idea of countervailing powers (in the absence of a global state). In the absence of a real separation between the three powers, the aggregation of Knowledge, Will and Power could ensure a sort of rebalancing, with each actor having a role in the elaboration and application of norms, to the extent that scientists’ independence and competence are respected and civic actors’ impartiality is guaranteed. Hence the importance of regulating possible conflicts of interest.

In short, it is no longer a question of separating powers, but of combining Knowledge and Will, which, in the face of economic or political Powers, or both, are the real embodiments of a community emerging from a moving law.

## V. Conclusion: thinking a moving law

Clearly, the law is moving: this is why the emerging normative phenomena cannot be conceived in light of the “pyramid of norms” metaphor alone. Despite the pillars, the foundations, the fundamental rights, we have entered a zone of turbulence, one that is inherently unstable. Of course, the metaphor of networks better reflects horizontality (networks of cities, of judges) than the metaphor of the pyramid,^13^ but it does not sufficiently express the growing instability that characterises our societies. Hence the metaphor of clouds and winds.^14^ Beyond the usual problems of translation (the rule of law is not synonymous with *état de droit*, human rights can refer to both the State subject to the law and the State that makes laws, common law does not have the same meaning as *droit commun*, etc.), the “founding concepts” should be replaced by “transformative processes”. From then on, little by little, the meanings of words are surreptitiously subverted: this is how sovereignty that was intended to be “solitary” could become “solidary”.

To sum up, one cannot choose between sovereignism and universalism; nor restrict legal systems to a hierarchical and binary logic; nor admit the appropriation of global common goods by States or TNCs; nor transpose the separation of powers on the scale of a world government; nor think of the global community as a community of memory. This is why the jurist must be creative and the law innovative. Of course, it is not a question of giving free rein to an unbridled imagination, but simply of going off the beaten track, because reality no longer passes through it. It involves a complexity that could paradoxically strengthen justice and new narratives of anticipation that should help to balance mere force.

To achieve this, we will have to change our cardinal points. In this confused world, there is no longer a North Pole, in the sense that it is impossible to choose among the adverse winds of globalisation. But we can imagine an unusual compass.^15^ At the centre, generated by the spiral of legal humanisms, an octagonal receptacle collects water, the symbol of life, where the regulatory principles reconciling the headwinds of globalisation meet. Plunged into this receptacle, the plumb line of good governance would stabilise disorderly movements without immobilising this world in motion.

Thus, inspired by the “imagining forces of law”,^16^ the jurist can try to respond to Pascal’s disillusioned observation in the seventeenth century that “since justice could not be strengthened, force was justified, so that justice and force might be together and peace might exist, which is the sovereign good”. If the spiral of humanisms strengthened justice, the octagon of regulatory principles would balance force. This does not mean, however, that we should adhere to the utopian dream of the two Ks: the “Great Peace” of the Chinese classics, taken up at the end of the nineteenth century by the jurist Kang Youwei; and the “Perpetual Peace” of the philosopher Immanuel Kant in the eighteenth century. In a more modest way, the goal is to put in place devices of appeasement, making peace with the earth.

## Footnotes

1

M Delmas-Marty , Towards a Truly Common Law: Europe as a Laboratory for Legal Pluralism (Cambridge, Cambridge University Press 2002 ); M Delmas-Marty , Ordering Pluralism: A Conceptual Framework for Understanding the Transnational Legal World (Oxford, Hart Publishing, CERDIN, coll French Studies in International Law 2009) p 175 .

2 See M Delmas-Marty , M Pieth and U Sieber (eds), Les chemins de l’harmonisation pénale (Paris, Société de législation comparée 2008) vol 15, p 423 et seq.

3
ibid, p 425.

4

F Jacob , La logique du vivant (Paris, Gallimard 1976).

5

A Garapon , Les “nouvelles routes de la soie”: La voie chinoise de la mondialisation, IHEJ, 2016 <http://ihej.org/wp-content/uploads/2016/11/La_route_de_la_soie.pdf>.

6

M Delmas-Marty , Le flou du droit (Paris, PUF 1986) p 336 (2nd edn 2004); M Delmas-Marty , Les forces imaginantes du droit (II), Le pluralisme ordonné (Paris, Seuil 2006) p 314 .

7

A Garapon and P Servan-Shreiber (eds), Deals de justice – Le marché américain de l’obéissance mondialisée (Paris, PUF 2014).

8 L. n° 2017-399, 27 March 2017 relating to the duty of vigilance of parent and client companies: JO 28 March 2017.

9

PL Périn , “Intérêt social élargi: un nouveau Pacte pour l’entreprise”, (2019) La Revue des Juristes des Sciences Po 16 .

10

M Delmas-Marty , Les forces imaginantes du droit (IV), Vers une Communauté de valeurs (Paris, Seuil 2011).

11

M Delmas-Marty , Aux quatre vents du monde – Petit guide de navigation sur l’océan de la mondialisation (Paris, Seuil 2016).

12

M Delmas-Marty and J Vervaele (eds), The Implementation of the Corpus Juris in the Member States: Penal Provisions for the Protection of European Finances, 4 vols (Cambridge, Intersentia 2000).

13 See F Ost and M van de Kerchove , De la pyramide au réseau? Pour une théorie dialectique du droit (Burssels, Publications des facultés universitaires Saint-Louis 2002).

14 Delmas-Marty, supra, note 11; M Delmas-Marty , Sortir du pot au noir: l’humanisme juridique comme boussole (Paris, Buchet Chastel 2019).

15 See M Delmas-Marty , Une boussole des possibles. Gouvernance mondiale et humanismes juridiques (Paris, Éditions du Collège de France, collection “Leçons de clôture” 2020).

16 See M Delmas-Marty , Les forces imaginantes du droit (Paris, Seuil 2004–2011).

